# *Macrophomina phaseolina:* A Phytopathogen Associated with Human Ocular Infections—A Case Report of Endophthalmitis and Systematic Review of Human Infections

**DOI:** 10.3390/jcm14020430

**Published:** 2025-01-11

**Authors:** Panagiotis Toumasis, Georgia Vrioni, Ioanna Gardeli, Aikaterini Michelaki, Maria Exindari, Maria Orfanidou

**Affiliations:** 1 Postgraduate Master Program “Ocular Surgery”, School of Medicine, Aristotle University of Thessaloniki, 54124 Thessaloniki, Greece; 2 Department of Microbiology, Medical School, National and Kapodistrian University of Athens, 11527 Athens, Greece; 3 Department of Ophthalmology, General Hospital “G. Gennimatas”, 11527 Athens, Greece; 4 Department of Clinical Microbiology, General Hospital “G. Gennimatas”, 11527 Athens, Greece; 5 Department of Microbiology, School of Medicine, Aristotle University of Thessaloniki, 54124 Thessaloniki, Greece

**Keywords:** endophthalmitis, fungi, fungal infections, human infection, *Macrophomina phaseolina*, ocular infection, phytopathogen, plant pathogen

## Abstract

**Background**: *Macrophomina phaseolina* is an important phytopathogenic fungus affecting over 500 plant species worldwide. However, this fungus rarely causes disease in humans. **Methods**: We reported the first case of endophthalmitis due to *M. phaseolina*, describing microbiological diagnostic approaches. Also, we performed a systematic review of human infections by this plant pathogen in literature. We searched PubMed, Scopus, and Web of Science databases from inception to 31 December 2024. **Results**: Our case involved a male patient who presented with photophobia and pain in his right eye. His recent medical history revealed a superficial corneal injury caused by a metal burr three months prior, managed unsuccessfully by topical treatment and subsequent conjunctival flap surgery two months later. Ophthalmological and microbiological investigations, including microscopic examination, cultures, and DNA sequencing of ocular specimens, revealed *M. phaseolina* endophthalmitis. Despite intravenous and intravitreal antifungal therapy, the patient’s condition continued to worsen, eventually leading to enucleation. Regarding the literature review, we identified 12 additional cases of *M. phaseolina* human infections previously reported in literature. Overall, *M. phaseolina* was primarily associated with ocular infections (76.9% of cases), followed by skin infections and combined skin–joint infections. The majority of patients with *M. phaseolina* infection (63.6%) had no known immunosuppressive factors. Clinical outcomes were unfavorable in 46.15% of cases. **Conclusions**: *M. phaseolina* is an emerging cause of human infections, even in immunocompetent hosts, with a predilection for ocular infections. Further research is warranted to elucidate the pathogenesis of fungal infections caused by plant pathogens in humans.

## 1. Introduction

Phytopathogens, while predominantly affecting plants, might also have implications for human health. Several plant pathogenic fungi, bacteria, and viruses have been isolated from humans, primarily from immunocompromised individuals, post-surgical patients, or those with post-traumatic injuries [1]. Clinical phytopathogens are typically regarded as opportunistic pathogens that lack specificity for human or animal hosts, as they do not rely on host-specific virulence factors, none of which have been identified in these pathogens to date [1]. Although there is a growing body of evidence demonstrating the ability of specific fungal phytopathogens to infect humans and animals, the specific mechanisms through which these fungi cause diseases in animals remain largely unclear [1,2]. In recent years, the misuse and/or overuse of synthetic mycotic eradicants in agriculture has led to the emergence of highly virulent fungal strains [2]. Some of these strains have crossed kingdom barriers and are now causing resistant human mycoses [2].

*Macrophomina phaseolina* is a soil- and seed-borne fungal species belonging to the Botryosphaeriaceae family of the phylum Ascomyceta [3,4]. It is primarily known as an important plant pathogen responsible for massive yield losses, causing stem and root rot, charcoal rot, and seedling blight in over 500 plant species [3,4]. The fungus persists in the soil as sclerotia for many years and, under favorable environmental conditions, produces hyphae that infect and damage plant roots [4]. However, *M. phaseolina* is considered an opportunistic invasive pathogen with the potential to disarm plant, animal, and human immunities [5]. Since clinical phytopathogens, including Macrophomina, are generally regarded as opportunistic and lack host-specific virulence factors, it is plausible to assume that infections by the genus Macrophomina may result from complex interactions between fungal agents and host factors, including tissue susceptibility and immunological mechanisms. However, further studies are needed to validate this hypothesis.

Despite its well-documented role as a destructive pathogen in agriculture, *M. phaseolina* remains significantly understudied as an opportunistic pathogen in humans, with existing knowledge primarily derived from isolated and sporadic case reports. Here, we report the first case of endophthalmitis caused by *M. phaseolina*, which ultimately led to an unfavorable outcome, necessitating enucleation of the affected eye. Additionally, we provide a systematic review of all reported cases of human infection by this plant pathogen in literature. Our aim is to offer a comprehensive understanding of the impact of *M. phaseolina* on human health.

## 2. Case Presentation

A 78-year-old male patient of provincial origin presented to “G. Gennimatas” General Hospital of Athens with photophobia and intense pain in his right eye during the previous 10 days, partially alleviated by oral analgesics (Day 0). Initially, a thorough ophthalmological history was taken. Three months before, the patient had a superficial corneal injury caused by a metal burr during manual labor in his field. He visited the local hospital in his residence area and was prescribed topical treatment. However, due to a non-healing corneal ulcer, the patient underwent conjunctival flap surgery two months later. On admission to our hospital, he was wearing therapeutic contact lenses, and he was prescribed eye drops (cyclopentolate 1%, dexamethasone 0.1%, ofloxacin 0.3%, and tobramycin 0.3%), along with a regenerative/healing eye ointment containing dexpanthenol. Additional information from his medical history included heterozygous beta-thalassemia, coronary artery disease, hypertension, dyslipidemia, and smoking (25 pack-years).

The patient’s best corrected visual acuity (BCVA) was 18/20 in the left eye (OS) and light perception (LP) in the right eye (OD). The slit-lamp examination of the OS did not provide significant findings, while the OD exhibited edema and redness of the upper and lower eyelids, conjunctival congestion and hyperemia, and corneal melting, as well as approximately 1 mm of hypopyon. The posterior segment was not visible, while B-scan ultrasonography revealed low-amplitude mobile echoes, vitreous membranes, and thickening of the retina and choroid. Clinical suspicion for endophthalmitis was prompted.

Corneal scrapings and vitreous humor samples were obtained for microbiological analysis (Day 0). Local anesthetic eye drops (tetracaine 0.5%) were instilled to the affected eye in order to minimize ocular discomfort and facilitate the samples collection procedure. Corneal scrapings were obtained from the base and edges of the corneal ulcer while the patient was positioned at the slit-lamp, using a sterile Kimura spatula. For the collection of vitreous humor, a 23-gauge needle attached to a 1 mL tuberculin syringe was inserted through the pars plana region into the cavity, and 0.1 mL of vitreous humor was aspirated. Both samples were promptly placed on glass slides, with one set prepared for Gram staining and the other treated with 10% potassium hydroxide (KOH). Also, a portion of the samples was directly inoculated onto Sabouraud dextrose agar (SDA) with and without cycloheximide and incubated at 37 °C under ambient air conditions. Furthermore, another portion of the samples was sent for molecular testing using a panfungal real-time polymerase chain reaction (RT-PCR) assay.

The direct microscopy of the Gram-stained slides did not reveal any bacteria but indicated the presence of fungal hyphae, while the KOH preparation showed abundant, narrow, and septate fungal hyphae. Since the clinical picture, as well as the microscopy findings, was suggestive of fungal keratitis-induced endophthalmitis, pending the outcome of other microbiological tests, the patient immediately started treatment. Voriconazole 0.02% and amikacin eye drops were applied, and the intravenous administration of voriconazole and moxifloxacin was started. Simultaneously, an intravitreal injection of voriconazole and amikacin was performed.

The following day (Day 1), panfungal RT-PCR results revealed a positive detection of fungal DNA, with a sensitivity of approximately 100 copies and a specificity greater than 95%. The fungal etiology of the disease was confirmed, and the patient’s treatment remained unchanged.

After, 3 days (Day 4), both corneal and vitreous humor cultures grew gray–black colonies on SDA without cycloheximide, while no growth was observed on the plates containing cycloheximide. Lactophenol cotton blue preparation from the colonies showed brown-colored hyphae with thickened cells. Additionally, minimum inhibitory concentrations (MICs) of the antifungal drugs amphotericin B, voriconazole, isavuconazole, itraconazole, and posaconazole were 0.047, 0.032, 0.064, >32, and 1  μg/mL, respectively. During the aforementioned period (Days 0–4), the patient’s clinical condition did not show signs of improvement. A computed tomography (CT) scan was conducted, and the results revealed no direct evidence of extensive orbital infection. The findings suggested that the infection was localized and confined to the ocular structures. After consultation with infectious disease specialists, intravenous treatment was modified to amphotericin B and isavuconazole, while the topical instillation of voriconazole and amikacin continued, and vancomycin eye drops were added to mitigate the risk of secondary bacterial infections, which can complicate severe infections like endophthalmitis.

**Figure 1:**
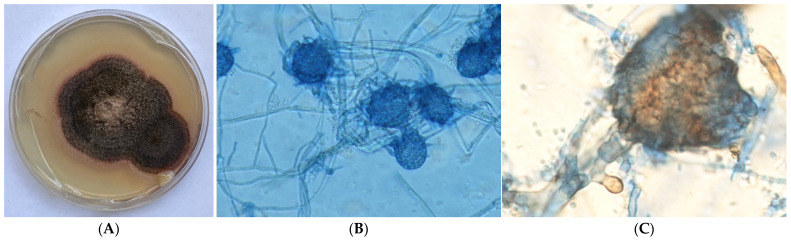
Macroscopic and microscopic characteristics of *M. phaseolina*. (**A**) Macroscopic appearance of the culture growth on potato dextrose agar; (**B**) microscopic appearance of the fungi with lactophenol cotton blue preparation at 1000× magnitude; (**C**) microscopic appearance of the sclerotium of *M. phaseolina* at 1000× magnitude.

A small piece of the colony from an SDA plate was inoculated on potato dextrose agar (PDA) in order to induce the development of diagnostic morphological features. Cultures on SDA were sent to the Microbiology Laboratory of the National and Kapodistrian University of Athens for matrix-assisted laser desorption/ionization time-of-flight (MALDI-TOF) mass spectrometry and DNA sequencing. The next day (Day 5), the MALDI-TOF Biotyper MSP (BRUKER) did not yield any results. After 2 days (Day 7), the fungal culture on PDA exhibited fast-growing, grayish-black colonies (Figure 1). Microscopic examination revealed the presence of sclerotia within these colonies (Figure 1). Although this data helped narrow down the range of potential pathogens, confirmation was required through molecular identification assays.

The patient’s condition continued to worsen (Days 4–7), showing no response to the dual antifungal treatment. Given the aggressive nature of the infection, the medical team determined that enucleation was necessary to prevent further spread of the infection, alleviate pain, and reduce the risk of systemic dissemination. The procedure was uneventful, and the patient was closely monitored postoperatively (Day 7). There was no evidence of fungal elements in the surrounding orbital tissues, suggesting that the infection was contained within the eye. However, systemic antifungal therapy with intravenous amphotericin B and isavuconazole was continued to ensure the eradication of any potential residual fungal infection. After 3 days (day 10), the patient was discharged with a clearly improved clinical condition and instructions to take oral isavuconazole 100 mg twice daily for 4 weeks.

After 4 days (Day 11), the causative agent was genotypically confirmed to be *M. phaseolina*. The procedure was initiated by scraping fungal growth from the SDA plate. The fungal material was then subjected to DNA extraction using the Maxwell cartridge in the platform (Maxwell^®^ RSC Blood DNA Kit (Promega, Madison, USA). For molecular identification, the internal transcribed spacer (ITS) region of the ribosomal DNA was amplified using the primers ITS4 (5′-TCCTCCGCTTATTGATATGC-3) and ITS5 (5′-GGAAGTAAAAGTCGTAACAAGG-3′). PCR amplification was performed using Biometra UNO II Thermoblock Thermal Cycler w/48 Block for 0.2 mL tubes as follows: 35 cycles of denaturation at 95 °C for 60 s, annealing at 56–58 °C for 90 s, and extension at 72 °C for 120 s and, finally, one cycle of denaturation at 95 °C for 60 s, annealing at 56–58 °C for 90 s, and extension at 72 °C for 300 s. The amplicon was electrophoresed on 2% agarose gel, stained with ethidium bromide, and visualized on the Biorad Universal Hood II Gel Doc System. Sequence analysis was performed using the BLAST bioinformatics tool. The resulting sequences were compared with reference sequences in GenBank, and the isolate was identified as *M. phaseolina* with a sequence similarity of 100%. The sequence was deposited in the GenBank database with accession number PQ421452.

**Figure 2:**
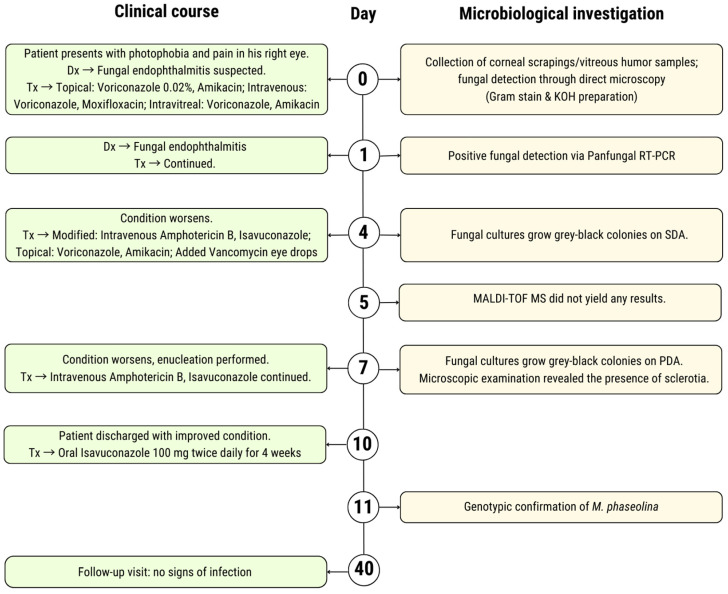
Timeline of clinical management and microbiological investigation of the patient. [Abbreviations: Dx = diagnosis, Tx = treatment, RT-PCR = real-time polymerase chain reaction, SDA = Sabouraud dextrose agar, and PDA = potato dextrose agar].

One month after the discharge (Day 40), the patient returned for a follow-up appointment. He reported a significant improvement in overall well-being, with no systemic symptoms or signs of recurrent infection. A timeline summarizing the clinical course and microbiological investigations is provided in Figure 2.

## 3. Review of the Literature

### 3.1. Methods

#### 3.1.1. Search Strategy

This systematic review followed the Preferred Reporting Items for Systematic Reviews and Meta-Analyses (PRISMA) guidelines [6]. An electronic search was conducted using the PubMed, Scopus, and Web of Science databases to identify studies published from their inception until 2024. The initial search was performed on 4 September 2024 and subsequently updated on 31 December 2024. The search string utilized was “(Macrophomina phaseolina) AND ((human) OR (patient))”. Additionally, in order to reduce the risk of missing data, a review of the references of each selected study was performed. The review process was independently performed by two authors (P.T. and G.V.), while any disagreements regarding the inclusion of the studies were resolved by a third author (M.E.).

#### 3.1.2. Eligibility Criteria

All observational studies, case series, or case reports referring to *M. phaseolina* infection in humans were included. There were no limitations based on language, geography, or time period. Studies with the following criteria were excluded: (1) secondary research studies (such as reviews or meta-analyses), editorials, and articles not reporting primary research results and (2) studies not performed on humans.

#### 3.1.3. Data Items

The primary study outcomes were to record (a) the epidemiology and clinical profile of patients with *M. phaseolina* infection and (b) the type of *M. phaseolina* infections included in literature. Secondary objectives focused on (a) characterizing the clinical features of different infection types, (b) gathering microbiological data on *M. phaseolina* infections, (c) reviewing treatment approaches, and (d) assessing patient outcomes.

#### 3.1.4. Data Synthesis

Two authors (P.T. and G.V.) extracted the data from each eligible study into a Microsoft Excel template. Extracted data included study type, country and year of publication, patient demographic data (gender and age), patient’s comorbidities, site of infection and relevant medical history, antifungal susceptibility data, treatment, and outcomes.

### 3.2. Results

#### 3.2.1. Study Selection Process and Study Characteristics

**Figure 3:**
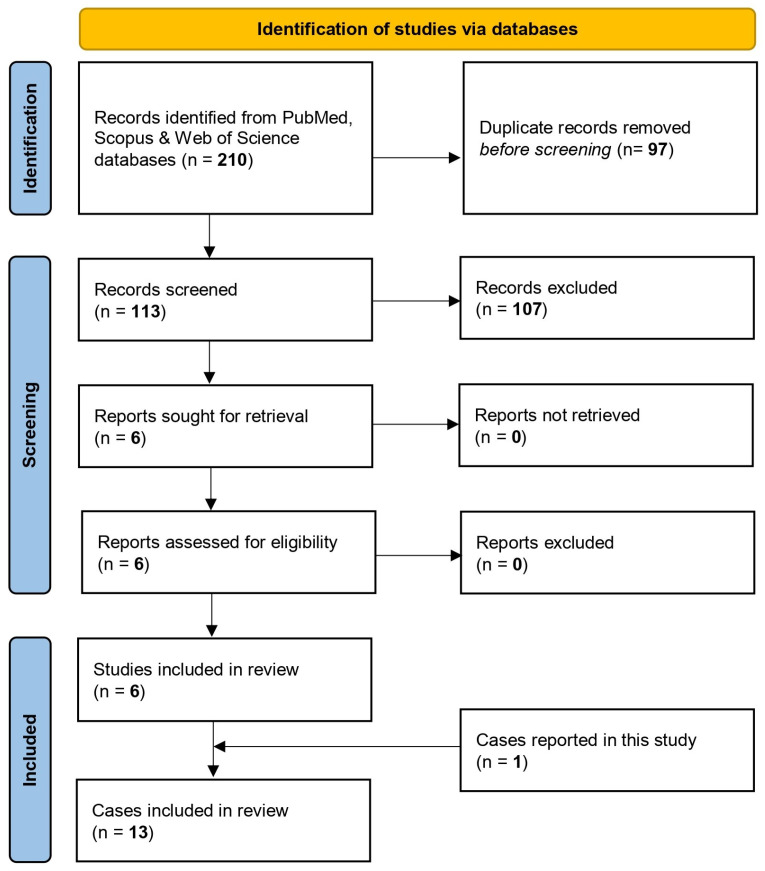
Flow chart showing the steps of the search and selection process of the systematic review.

A total of 210 studies were retrieved from the research in the electronic literature databases; 97 were duplicate entries and were removed using EndNote X8. After thoroughly screening titles and abstracts of the remaining studies, 107 were excluded either because their subject matter did not align with the purpose of the review, focusing on aspects of *M. phaseolina* infection in plants, or because they met other exclusion criteria. A careful study of the entire text of the remaining six studies revealed that all of them met the inclusion criteria and were therefore included in this systematic review [7,8,9,10,11,12]. Among the included studies, four were case reports [7,9,10,11], and two were laboratory-based observational studies that provided clinical data on patients with *M. phaseolina* infection [8,12]. Manual screening of the references from the included articles, as well as a search on Google Scholar, did not identify any additional studies. The studies that resulted from the electronic search evaluated data from 12 patients. Including our case, a total of 13 cases were finally included in this systematic review. The flowchart is presented in Figure 3.

**Figure 4:**
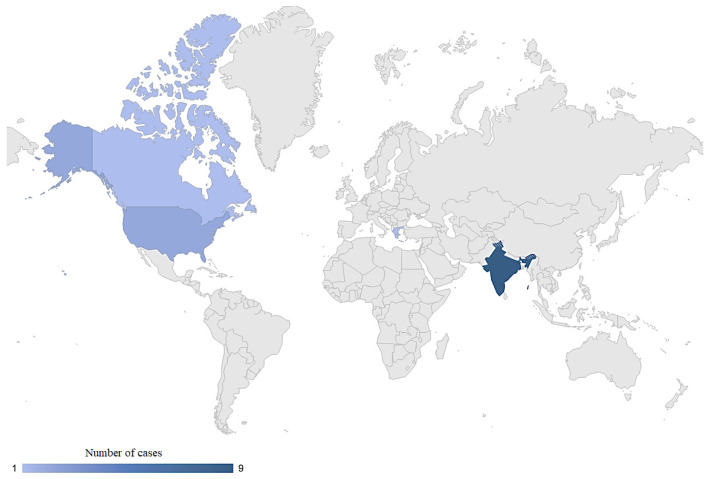
Map of countries where cases of *M. phaseolina* infection were documented.

The countries where cases of *M. phaseolina* infection have been documented are Canada, India, the USA, and Greece (Figure 4).

#### 3.2.2. Clinical Profile of Patients with *M. phaseolina* Infection

Details of all cases (13 patients) are provided in Table 1. Age and sex of patients were available for eleven patients; the median age was 52 years (IQR 42–65), and six (54.6%) were male. Human infections caused by *M. phaseolina* were predominantly ocular, accounting for 76.9% (10 of 13 cases), which included nine cases of keratitis and one case of endophthalmitis. Skin infections were observed in 15.4% of cases (two cases), while a combined skin and joint infection was reported in 7.7% (one case). The acquisition of *M. phaseolina* ocular infection was linked to ocular trauma or injury by a foreign body in 70% (7 of 10 cases). Immune status was available for eleven cases; four (36.4%) cases were immunocompromised, including one transplant recipient under immunosuppressive therapy, one oncology patient, one diabetic patient, and one patient with chronic kidney disease; and eleven (63.6%) cases had no comorbidities with direct immunosuppressive impact. All cases (100%) that were reported as immunocompetent had ocular infections. Identification methods of *M. phaseolina* at the species level was available for 11 cases, all confirmed using molecular techniques, including DNA sequencing techniques and/or PCR assays of a specific gene sequence. Data on the in vitro antifungal susceptibility of *M. phaseolina* were available in eight patients. The antifungal agents used for *M. phaseolina* infections included voriconazole, natamycin, ketoconazole, posaconazole, amphotericin B, and isavuconazole. Regarding the in vivo response of the fungus to specific antifungal agents, 50% (two out of four) of the cases treated exclusively with voriconazole failed to respond to therapy and required a change in treatment, despite antifungal susceptibility testing indicating that their *M. phaseolina* strains were susceptible to the drug. Regarding clinical outcomes of *M. phaseolina* infections, 46.15% (six cases) had unfavorable outcomes, despite antifungal treatment, requiring surgical intervention. All cases with unfavorable outcomes were associated with ocular infections.

## 4. Discussion

*M. phaseolina* is known and well described as an important phytopathogenic fungus. Our systematic review of 13 human cases, including the currently described one, underscores its emerging role as a pathogen in human infections the last 15 years. The most common form of *M. phaseolina* infection in humans involves the eye, with 76.9% of cases manifesting as either keratitis or endophthalmitis. Our case is the first and only documented case of endophthalmitis caused by *M. phaseolina*, highlighting the invasive properties of this phytopathogen when infecting humans, as well as the challenges associated with its medical treatment.

Human infections caused by plant pathogens are typically associated with immunocompromised patients [1]. However, only 36.4% of the included cases had underlying immunosuppressive conditions. The majority of patients with *M. phaseolina* infection (63.6%) were individuals with no known immunosuppressive factors. This fact may indicate that the pathogen possesses a higher degree of virulence, enabling it to infect even otherwise healthy individuals.

The clinical manifestations of *M. phaseolina* infections do not appear to be pathognomonic for this pathogen. Instead, the observed signs and symptoms of inflammation in affected tissues (eye and skin) are comparable to those caused by other fungal pathogens. This lack of specificity highlights the challenge of diagnosing *M. phaseolina* infections based solely on clinical presentation.

Diagnosis of *M. phaseolina* infections in humans is based on specialized microbiological techniques. Traditional fungal identification methods, while valuable, may not be sufficient, especially given *M. phaseolina*’s morphological similarities to other fungi. Although the presence of sclerotia observed under microscopy might be a distinct phenotypic feature, it is not pathognomonic for *M. phaseolina* [13]. Molecular methods were reported in 90.9% of cases. However, molecular testing for *M. phaseolina* can be complex and may require multiple methods to achieve accurate identification. BLAST analysis of ITS sequences might suggest one species, but a more refined analysis of type material can lead to a different conclusion. In a small case series, while initial results of all fungal isolates pointed towards *M. phaseolina*, when restricted to sequences from type material, they aligned with *M. pseudophaseolin* [12]. Phylogenetic analysis was unable to distinguish between the two species, placing them in the same clade, while a PCR assay targeting the specific gene sequence, namely MpCal (encodes calmodulin), confirmed that all isolates were *M. phaseolina*. Another aspect about molecular testing was set in our case. We made an initial attempt to identify *M. phaseolina* using MALDI-TOF MS; however, it did not yield any results. Although MALDI-TOF MS is a rapid and reliable method for fungal identification, it relies on a reference database of protein profiles [14]. It seems that *M. phaseolina* is not well represented or absent in the database used, thus leading to inefficiency of identification.

Similarly with other fungal infections, treatment of *M. phaseolina* infections also remains a clinical challenge. Clinical outcomes were poor in 46.15% (six patients), despite susceptibility results showing in vitro sensitivity. It is noteworthy that two out of four (50%) patients who were treated with voriconazole alone, including our patient, did not respond to voriconazole treatment, despite antifungal susceptibility tests showing *M. phaseolina* strains to be susceptible [7]. The site of infection plays a crucial role; the patient with a skin infection may have experienced poor drug penetration in the affected area, while the patient with endophthalmitis likely faced challenges with voriconazole reaching therapeutic levels in the eye. Furthermore, the inconsistency between in vitro susceptibility and clinical outcomes could be attributed to intrinsic resistance mechanisms of *M. phaseolina*. Fungi might present resistance mechanisms that are not detected through standard susceptibility testing, such as efflux pump overexpression or enzymatic degradation of the drugs [15]. The intraocular penetration of antifungal drugs can be variable, and the concentrations achieved in the vitreous humor may not have been sufficient to effectively eradicate the infection [16]. The corneal avascularity, as well as protective barriers and anatomical characteristics of the eye limit, lessen the effectiveness of topical or systemic and oral drugs. Moreover, the very low ocular bioavailability of topical drugs (less than 5%) makes the efficient treatment of several ocular diseases very difficult [17,18] Recently, some studies have shown that specific nanocarriers can interact with the ocular mucosa, thereby increasing the retention time of the associated drug onto the eye, as well as its permeability across the corneal and conjunctival epithelia [19,20]. Therefore, skilled ophthalmological expertise is required to track and monitor every development in disease etiology.

One of the key limitations of this review is the potential bias due to underreporting of cases, as rare infections such as *M. phaseolina* in humans are often not documented in literature. Additionally, publication bias may influence the representation of these infections, as cases that are more clinically significant or result in more severe outcomes are more likely to be reported, potentially skewing the findings of this review.

## 5. Conclusions

*M. phaseolina*, a common plant pathogen, is an uncommon but emerging cause of human infections. This case report of endophthalmitis highlights the pathogen’s potential to cause severe ocular infections with devastating outcomes, even despite aggressive treatment. The systematic review of previously reported human infections emphasizes the diverse clinical presentations, ranging from superficial skin infections to more invasive conditions like the one we described. However, *M. phaseolina* appears to be a fungal phytopathogen with a predilection for ocular infections in humans. Timely diagnosis remains a challenge due to its rarity in clinical settings and the absence of standardized treatment guidelines. However, early identification and appropriate antifungal therapy are critical for favorable outcomes. Given the growing number of human cases reported, it is essential for clinicians to be aware of *M. phaseolina* as a potential pathogen in vulnerable patients. Further research is warranted to elucidate the pathogenesis of fungal infections caused by plant pathogens in humans, identify evidence-based therapeutic strategies, and develop effective preventive measures to minimize its impact on clinical outcomes and public health.

## Figures and Tables

**Table 1:** Summary of reported cases of *M. phaseolina* infections in humans.

Case	Article (Year)	Country	Age (y)	Gender	Site(s) of Infection	Predisposing Factor	Comorbidities	Microbiological Method to Confirm *M. phaseolina* Infection	Antifungal Susceptibility—MIC	Antifungal Treatment	Outcome Regarding *M. phaseolina* Infection	REF.
1	Tan et al. (2008)	Canada	31	M	Skin and joint (Left great toe)	Unknown	Hypertension End-stage renal disease (Renal transplantation two months prior to the infection. Under immunosuppression regimen.)	DNA sequencing	AΜΒ = 0.06 μg/mL VRZ = 0.015 μg/mL KCZ = 0.03 μg/mL CAS = 0.015 μg/mL ITZ = 0.015 μg/mL FLU = 0.12 μg/mL 5-FC = 4 μg/mL	Oral VRZ 200 mg twice daily	Favorable (Symptoms improved) However, unfavorable overall outcome (Death of invasive infection by the related pathogen, *Scytalidium dimidiatum*)	[7]
2	Bagyalakshmi et al. (2008)	India	N/A	N/A	Eye (Keratitis)	Unknown	N/A	DNA sequencing	N/A	AMB eye drops	Favorable (Ocular lesion resolved completely)	[8]
3	//	//	N/A	N/A	Eye (Keratitis)	Trauma	N/A	DNA sequencing	N/A	AMB eye drops	Favorable (Ocular lesion resolved completely)	//
4	Srinivasan et al. (2009)	USA	6	F	“Skin (Right medial malleolus)”	Unknown	Acute myeloid leukemia	DNA sequencing	AΜΒ = 0.25 μg/mL VRZ = 0.5 μg/mL PZC = 1 μg/mL TER = 0.06 μg/mL CAS = 1 μg/mL	VRZ (route of admission not mentioned) → no improvement → change to PZC (route of admission not mentioned)	Favorable (Dramatic improvement with PZC; complete healing with central scarring within 3 weeks)	[9]
5	Premamalini et al. (2012)	India	70	F	Eye (Keratitis)	Trauma	Anemia Chronic kidney disease	DNA sequencing	N/A	Initially: Natamycin eye drops hourly, oral KCZ 200 mg twice daily After identification: Natamycin eye drops two hourly, oral VRZ 200 mg twice daily	Favorable (Ocular lesion and symptoms resolved completely)	[10]
6	Schwartz et al. (2020)	USA	42	M	Skin (soft tissue mass on the right dorsal foot)	Unknown	Diabetes mellitus	N/A	N/A	Oral VRZ 300 mg twice daily for 1 day and then 200 mg twice daily for 2 weeks	Favorable (The mass resolved completely)	[11]
7	Ahirwar et al. (2022)	India	46	M	Eye (Keratitis)	Foreign body	No	PCR assay of MpCal gene	Ν/A	NAM eye drops, VRZ eye drops, oral KCZ 200 mg	Favorable (Ocular lesion resolved completely)	[12]
8	//	//	58	M	Eye (Keratitis)	Unknown	No	PCR assay of MpCal gene	N/A	NAM eye drops, KCZ 200 mg	Unfavorable (Therapeutic penetrating keratoplasty)	//
9	//	//	46	F	Eye (Keratitis)	Unknown	No	PCR assay of MpCal gene	AMB = 2 μg/mL VRZ = 0.1 μg/mL PZC = 0.1 μg/mL KCZ = 16 μg/mL CAS = 0.03 μg/mL NAT = 2 μg/mL	NAM eye drops, KCZ 200 mg	Unfavorable (Therapeutic penetrating keratoplasty)	//
10	//	//	52	F	Eye (Keratitis)	Foreign body	No	PCR assay of MpCal gene	AMB = 8 μg/mL VRZ < 0.01 μg/mL PZC < 0.01 μg/mL KCZ = 1 μg/mL CAS = 0.03 μg/mL NAT = 4 μg/mL	NAM eye drops, KCZ 200 mg	Unfavorable (Therapeutic penetrating keratoplasty)	//
11	//	//	65	M	Eye (Keratitis)	Trauma	No	PCR assay of MpCal gene	AMB = 4 μg/mL VRZ = 0.03 μg/mL PZC = 2 μg/mL KCZ = 16 μg/mL CAS = 4 μg/mL NAT = 2 μg/mL	NAM eye drops, KCZ 200 mg	Unfavorable (Therapeutic penetrating keratoplasty)	//
12	//	//	56	F	Eye (Keratitis)	Foreign body	No	PCR assay of MpCal gene	AMB = 4 μg/mL VRZ = 2 μg/mL PZC = 2 μg/mL KCZ = 2 μg/mL CAS = 8 μg/mL NAT = 2 μg/mL	NAM eye drops, KCZ 200 mg	Unfavorable (Therapeutic penetrating keratoplasty)	//
13	Toumasis et al. (2024)	Greece	78	M	Eye (Endophthalmitis)	Foreign body	Heterozygous beta-thalassemia Coronary artery disease Hypertension Dyslipidemia	DNA sequencing	AΜΒ = 0.047 μg/mL VRZ = 0.032 μg/mL ISAV = 0.064 μg/mL ITZ > 32 μg/mL PZC = 1 μg/mL	VRZ eye drops, IVT and IV for 4 days → no improvement → change to AMB/ISAV IVT and IV for 3 days → no improvement → enucleation After enucleation: IV AMB/ISAV for 3 days After discharge: oral ISAV for 4 weeks	Unfavorable (Enucleation of the affected eye)	[*]

Abbreviations: N/A = not available, y = years, M = male, F = female, MIC = minimum inhibitory concentration, IV = intravenous, IVT = intravitreal, AMB = amphotericin B, ISAV = isavuconazole, ITZ = itraconazole, VRZ = voriconazole, CAS = caspofungin, PZC = posaconazole, TER = terbinafine, KCZ = ketoconazole, NAT = natamycin, FLU = fluconazole, 5-FC = 5-fluorocytosine. * Present study.
